# Supplementary material for: “It becomes more difficult when people don’t empathize with us”: COVID-19-related stigmatization experienced by survivors in Nepal
Source: PLoS One. 2026 Mar 6;21(3):e0344123. doi: 10.1371/journal.pone.0344123 (PMC12965614; doi:10.1371/journal.pone.0344123)
Supplement: S1 File — (DOC) [file pone.0344123.s001.doc]

Supporting information file 1: Additional information

S Table 1: **Characteristics of study participants included in the in-depth interviews**

| **Characteristics** | **Categories** | **N (%)** |
| --- | --- | --- |
| **Age** | 18-30 yrs. | 4 (26.6) |
|  | 31-45 yrs. | 7 (46.6) |
|  | 46 and above | 4 (26.6) |
| **Sex** | Female | 7 (46.6) |
|  | Male | 8 (53.3) |
| **Education** | Primary level | 2 (13.3) |
|  | Secondary level | 3 (20) |
|  | Higher secondary and above | 10 (66.6) |
| **No of family members** | 1 to 3 | 7 (46.6) |
|  | 4 to 6 | 7 (46.6) |
|  | 7 and above | 1 (6.66) |
| **Occupation** | Student | 3 (20) |
|  | Employed | 11 (73.3) |
|  | Unemployed | 1 (6.66) |

S Table 2: :Different subthemes/codes on stigma and discrimination and their supporting verbatim

| **Subthemes/codes** | **Supporting verbatim** |
| --- | --- |
| Stigma experience among COVID-19 survivors and their families | *People got infected, and were in home isolation, and even then others called the society and the police and the infected were kicked out of the house. Even they weren’t economically stable, they were force out of the home isolation to go to a hotel. Many people were talking about how they were pressurized by the society even when they were in home isolation. [Male, 45-50 yrs.; educated and employed]*  *After I become recovered from COVID, people still thought that I have virus in my body and try to get far from me till 15 days or 1 month more also they think that although people was recovered, it takes more months to completely to go virus from human body. [female, 25-30 yrs.; educated and employed]* |
|  | *There was a lot of discrimination. Near to my home, due to lack of care even he was unable to boil water by himself. At the time of taking him hospital, he died. And support is also found a lot in the society. [Male, 25-30 yrs.; educated and employed]*  *From community hmm, at that time I didn’t get support and help for about 2,3 months. The term social stigma was taken totally in negative way like only being visible to the infected person also may transmit them. [P02: Male, 35-40 yrs.; educated and employed]*  *There was no such discrimination inside the house but outside the community people do discriminate. “He/she got COVID we should not walk by that house, we should not use that road. He/she this and that” [P03: Male, 50-55 yrs.; educated and employed]* |
|  | *So what happed is; since we were infected, people suspected the other non- infected 4 members too and in village area and people do have different way of perceiving things so they used to turn their face around while walking through our house. They felt that discrimination. No one cared to come and ask how the situation is and they felt isolated even though they were not infected. They did share those things to me and I felt bad about it.[P10: Male, 45-50 yrs; educated and employed]*  “*They report in police station that  they gone outside from home ,they walked in front of our house etc…..They don't come where we are  they   ask us  this this people  said that that and I said yes this this happened  I’m positive but we didn't affect anyone, we walked only in road to go for cutting grass in the ( Pakhama ) And all people discriminate us when we go , walk in outside , no one come and my  child are small can't cook and eat  and they manage to cook and eat hardly and lived ,  neighbor are not care about it they didn't see us  , what we eat  etc. they do discriminate” .[Female, 35-40 yrs; illiterate and unemployed]* |
|  | *I thought that no one will come to look after me and if anything will happen to me then who will take care of my child.* |
| Stigma practices associated with COVID-19 | *Some of my neighbors don’t touch anyone of our house and maintain distance from us. They used to close their door whenever they saw me. [P13: Female, 25-30 yrs; illiterate and unemployed]*  *It’s mostly seen in many places like using bamboo to block road with “no entry” sign, not letting to use that road saying that’s infected with corona, and that area used to be isolated and silent. For example a doctor got corona in Birat nursing home in Biratnagar and with him all of the staffs and the area got sealed and at that time we feared and didn’t used that road that we might get infected too. And that’s what I saw… and nothing more*. *[P03: Male, 50-55 yrs.; educated and employed]*  *“They are infected by corona we should not touch them or not even be near them at all” you know in a dominating way. It felt dominating. [P05: Male, 40-45 yrs.; educated and employed]*  *We should not use that road there’s corona. Community people took covid patient as someone not worthy (xixi dhur dhur ko patro).* |
| Well-being of persons with COVID-19 infection | *“People even didn’t allow to stay in the home to infected people like health worker who were working in a hospital as a doctor nurse. As they had a high chance of infection. So, people were scared from them and as well as from front line health workers; thinking they will spread the virus” (ID 12: 18- 30 yr., Male)*  *As I recovered, I sometimes felt that my neighbours and shopkeeper who knew I was infected didn’t try to come close to me. They would stay far from me, however they would talk to me. (ID 01: 20- 25 yr., Male, student)*  *And they feared that it affects their family when they walk by that road. They had this bad thoughts that they will get infected and their family would be in danger.*  *[P03: Male, 50-55 yrs.; educated and employed]* |
| Fragile interpersonal trust and support | *During that time the main problem was people were scared of being taken by the police and used to hide the fact that they have the disease. If any case was found the area would have been sealed, people would hesitate to go near the patients and even the whole family would have the faced the same.[P10: Male, 45-50 yrs.; educated and employed]*  *In hospital, they told me that I bring covid by visiting here and there. But I had never gone anywhere I just go on duty and come back home. [P13: Female, 25-30 yrs; illiterate and unemployed]*  *It’s mostly seen in many places like using bamboo to block road with “no entry” sign, not letting to use that road saying that’s infected with corona, and that area used to be isolated and silent. For example a doctor got corona in birat nursing home in biratnagar and with him all of the staffs and the area got sealed and at that time we feared and didn’t used that road that we might get infected too. And that’s what I saw… and nothing more. [P03: Male, 50-55 yrs.; educated and employed]* |
| Fear of transmission/death of disease | *I think that people thought that this disease is an epidemic disease and it has come worldwide and many have lost their lives also and everyone wants to live and they get scared of getting infected. So, to save their lives they were being secure*. *Of course there will be different behavior. They were also fear getting transmitted so they talk from distance, some people said that it will be transmitted even when talking and closed doors. [P11: female, 45-50 yrs.; educated and employed]*  *While I have to add on this, I think the fear also plays its part. The fear of getting infected is everywhere, there are no guaranteed treatment, that makes the discrimination more worse. [P03: male, 35-40 yrs.; educated and employed]*  *Because in which way the death rates are increasing in west, in India, also in our own country, that’s why people have that fear and such feelings they just don’t say or show. They feel that once corona gets you, you die. [P03: Male, 50-55 yrs.; educated and employed]* |
| Misconception/ lack of awareness / Limited information about COVID-19 | *Discrimination should not be existed but due to the lack of awareness discrimination occurs nothing more than that. Safety should exist than the discrimination. Applying of the health safety measures like making some distance. Its ok to not hug or being too close. [P12: male, 25-30 yrs.; educated and employed]*  *Oh…to be honest, it is all about education. Had society been well informed and educated, such discriminations wouldn’t be there. I know ummm, for a disease like corona, it is necessary we keep some distance, but there is a standard protocol of 3 metres or 6 metres. More than that, is done because of lack of proper information. [P02: Male, 35-40 yrs.; educated and employed]*  *I think its lack of awareness (aagyanta). They don’t think about what's happening to others and how they are behaving, someday that can happen to them as well. They don’t have such a realization. That’s what I think. [P05: Male, 35-40 yrs.; educated and employed]*  *This is all because of lack of education and ignorance. Some are also happening because of people thinking that they will not be infected with corona. This is also due to a lack of education…..This should be known by people themselves that we should not discriminate against others, it is an only vulnerable disease if it is infected to him today then tomorrow we can also be infected, corona did not leave anyone. . [P06: Male, 55-60 yrs.; educated and unemployed]* |
