# Supplementary material for: “It becomes more difficult when people don’t empathize with us”: COVID-19-related stigmatization experienced by survivors in Nepal
Source: PLoS One. 2026 Mar 6;21(3):e0344123. doi: 10.1371/journal.pone.0344123 (PMC12965614; doi:10.1371/journal.pone.0344123)
Supplement: Attachment — Submitted filename: Rebuttal letter.pdf [file pone.0344123.s005.pdf]

<u>Response to Reviewer's comments –</u> PONE-D-24-58430R1

We thank the editor and reviewers for their time, careful assessment, and constructive feedback which have significantly improve the quality of our manuscript. All comments have been addressed thoroughly, and point-by-point responses to all the comments are provided below

1. **Reviewer #1: In one place, you say 'All participants in this study reported experiencing stigma from their families and community members, manifesting in various forms and intensities during their infection' and then "No participants reported direct discrimination from immediate family members". This is confusing and contradictory. If people have not faced stigma from family, then do not mention it as such in the first sentence.**

Response: Thank you for highlighting this inconsistency. We agree that the original wording was unclear and potentially misleading. We have revised the manuscript to clearly distinguish between stigma experienced from community members and experiences within the family context.

In the Results section, we now state that all participants reported experiencing stigma directed at themselves and, in some cases, their families from community members and neighbours following disclosure of their COVID-19 status. We further clarify that direct discrimination from immediate family members was largely absent, except one participant who described internalized stigma within the family context, where self-directed fear of infection shaped his behaviour rather than overt discriminatory actions. These clarifications are reflected consistently in both the Results and Discussion sections of the revised manuscript. (Line 216-223,429-435)

2. **The code you have, 'discrimination by family and friends, ' is also problematic, as you move on to say there was no discrimination. If this is the case, then term in as a 'Lack of discrimination by family and friends'. Please go through the codes and reword as appropriate.**

Response: Following this review, we refined the wording of several codes to better reflect the underlying data. Importantly, we removed any code that implied direct discrimination by immediate family members, as the data indicated that family-level stigma was limited to internalized stigma in one participant rather than overt discriminatory behaviour. As such, a separate code for family discrimination was not retained.

Under Theme 1, the revised codes now include: *internalized stigma due self-directed fear of infection, perceived stigma, strained social relationships, reduced social support, intersecting stigma, discrimination in community and workplace settings, and stigma directed towards family members of COVID-19 survivors*. These refinements are presented in Table 2. The revisions reflect improved clarity and precision in coding and do not alter the study results or their interpretation.

We appreciate the reviewer's thoughtful guidance, which has strengthened the conceptual clarity of the analysis.

3. **I do not think the authors have understood my previous comment about the need to discuss the findings of this study in relation to the current COVID-19-free context? Authors should address the context-specific nature of COVID-19 stigma, as it may now be almost non-existent. unlike the stigma surrounding, for example, HIV/AIDS or NTDs. This is because COVID-19-related stigma is likely to be temporary and does not exist now? Another angle could be how or whether these stigma experiences of these patients will affect help-seeking behaviors for other diseases as well? Will they have long-term socio-cultural effects? These need to be critiqued.**

Response: Thank you for this clarification. We agree that COVID-19-related stigma is context-specific and likely to be time-limited, and this is now explicitly addressed in the revised discussion section.
The discussion situates the findings within the early and acute phases of the COVID-19 pandemic, shaped by uncertainty, self-directed fear of infection, and restrictive public health measures. It acknowledges that such stigma may no longer be prominent in the current context. We also distinguish COVID-19-related stigma from more enduring forms of stigma, such as those associated with HIV/AIDS or neglected tropical diseases. Rather than implying persistence of COVID-19 stigma, the revised discussion frames the relevance of the findings in terms of lessons for future public health emergencies, including implications for risk communication, continuity of essential health services, and potential effects on help-seeking behaviours during future outbreaks. [Line 451-456, 401-409,479-483-511]

4. **The conclusion of this study needs to be thoroughly revised.**
In the Conclusion, the authors say "these findings emphasize the urgent need for comprehensive interventions to combat stigma and discrimination" but now that COVID is over, is the stigma still there? Is it still relevant to suggest something like that? What you need to suggest is how these findings can be used for future preparedness of such outbreaks. Again, I question the relevance of this sentence to the current context. The key recommendations include implementing mass education and awareness campaigns to improve knowledge about disease transmission, prevention, and control, establishing platforms for accessing accurate information, strengthening reporting systems, enhancing the capacity of health institutions, and developing robust social protection mechanisms to support individuals facing stigma.' Are people still not aware of COVID? Is this suggestion relevant to the current context?

Response: Thank you for highlighting this important issue. We agree that framing COVID-19-related stigma as ongoing would not be appropriate in the current context. In response, we have substantially revised the conclusion to remove language implying an urgent need to address ongoing COVID-19-related stigma.

The revised conclusion now explicitly acknowledges that COVID-19-related stigma has likely diminished in the post-pandemic period. Recommendations are no longer framed as current COVID-19 interventions but instead emphasize how the findings can inform future outbreak preparedness and response, including transferable lessons related to communication, health system capacity, and social protection during public health emergencies. (Line 476-489)

In response to the reviewer's emphasis on the post-pandemic context, we also reviewed the manuscript more broadly. We revised the abstract and introduction sections to ensure that the study is clearly framed as examining stigma during the acute phase of COVID-19, with relevance for future public health emergencies rather than the current COVID-19 context.

Reviewer #2: All the comments of the previous review have been addressed adequately by the authors in this submission.

Response: We thank the reviewer for their constructive comments, which have helped us substantially improve the quality and clarity of the manuscript.
