# Supplementary material for: “It becomes more difficult when people don’t empathize with us”: COVID-19-related stigmatization experienced by survivors in Nepal
Source: PLoS One. 2026 Mar 6;21(3):e0344123. doi: 10.1371/journal.pone.0344123 (PMC12965614; doi:10.1371/journal.pone.0344123)
Supplement: Attachment — Submitted filename: PONE-D-24-58430_ED.docx [file pone.0344123.s002.docx]

PLOS ONE

“It becomes more difficult when people don’t empathise with us": Exploring COVID-19 stigmatisation and its ecological impact on survivors in Nepal

--Manuscript Draft--

| Manuscript Number: | PONE-D-24-58430 |
| --- | --- |
| Article Type: | Research Article |
| Full Title: | “It becomes more difficult when people don’t empathise with us": Exploring COVID-19 stigmatisation and its ecological impact on survivors in Nepal |
| Short Title: | COVID-19 stigmatisation and its ecological impact |
| Corresponding Author: | Buna Bhandari  Tribhuvan University Institute of Medicine Kathmandu, Bagmati NEPAL |
| Keywords: | COVID-19; stigma; Discrimination; Nepal; Pandemic |
| Abstract: | The COVID-19 pandemic has significantly impacted individuals’ psychological wellbeing worldwide, with stigma emerging as a critical issue for survivors. In Nepal, stigma has further complicated the experiences of survivors, influencing their health, relationships, and social interactions. This study explores the multidimensional components of COVID-19-related stigma among survivors in Eastern Nepal. This qualitative study employed in-depth interviews with 15 COVID-19 survivors, identified from a prior survey, who reported experiencing stigma. Due to pandemic-related restrictions, interviews were conducted over the phone. Data were analysed thematically, guided by the socio-ecological framework. COVID-19 stigma was multifaceted, including social rejection, self-stigmatization, discrimination by family and friends, and labelling from community members. Survivors recounted forced quarantines, financial strain, and removal from homes by police. These experiences undermined trust, self-confidence, and mental well-being. Key drivers of stigma included fear of disease transmission, misinformation, and inadequate healthcare systems and policies that failed to address stigma effectively. Addressing COVID-19related stigma requires a comprehensive approach, including mass education campaigns to address myths, platforms for accurate information dissemination, and strengthening health systems. Interventions must also address systemic policy and healthcare infrastructure gaps to foster an inclusive and supportive environment for survivors. |
| Order of Authors: | Buna Bhandari, MPH,PhD |
|  | Poshan Thapa, MPH,PhD |
|  | Amit Timilsina, MPH |
|  | Rajiv Ranjan Karn, MPH |
|  | Haider Ali, MPH |
|  | Ashley Hagaman, MPH,PhD |
|  | Archana Shrestha, MPH,PhD |
| Additional Information: |  |
| Question | Response |
| Financial Disclosure  Enter a financial disclosure statement that describes the sources of funding for the work included in this submission. Review the [submission guidelines](http://journals.plos.org/plosone/s/submission-guidelines#loc-financial-disclosure-statement) for detailed requirements. View published research articles from [PLOS ONE](https://journals.plos.org/plosone/) for specific | Yes |

| examples.  This statement is required for submission and will appear in the published article if the submission is accepted. Please make sure it is accurate. |  |
| --- | --- |
| Funded studies Enter a statement with the following details: - Initials of the authors who received each award - Grant numbers awarded to each author - The full name of each funder - URL of each funder website - Did the sponsors or funders play any role in the study design, data collection and analysis, decision to publish, or preparation of the manuscript? |  |
| Did you receive funding for this work? |  |
| Please add funding details.   as follow-up to "Financial Disclosure  Enter a financial disclosure statement that describes the sources of funding for the work included in this submission. Review the [submission guidelines](http://journals.plos.org/plosone/s/submission-guidelines#loc-financial-disclosure-statement) for detailed requirements. View published research articles from [PLOS ONE](https://journals.plos.org/plosone/) for specific examples.  This statement is required for submission and will appear in the published article if the submission is accepted. Please make sure it is accurate. | The study was funded by Nepal Health Research Council provincial grant [Grant number 778]. The funders had no role in study design, data collection and analysis, and preparation of this manuscript. |
| Funded studies Enter a statement with the following details: - Initials of the authors who received each award - Grant numbers awarded to each author - The full name of each funder - URL of each funder website - Did the sponsors or funders play any role in the study design, data collection and analysis, decision to publish, or preparation of the manuscript? |  |
|  |  |

| Did you receive funding for this work?" |  |
| --- | --- |
| Please select the country of your main research funder (please select carefully as in some cases this is used in fee calculation).   as follow-up to "Financial Disclosure  Enter a financial disclosure statement that describes the sources of funding for the work included in this submission. Review the [submission guidelines](http://journals.plos.org/plosone/s/submission-guidelines#loc-financial-disclosure-statement) for detailed requirements. View published research articles from [PLOS ONE](https://journals.plos.org/plosone/) for specific examples.  This statement is required for submission and will appear in the published article if the submission is accepted. Please make sure it is accurate. | NEPAL - NP |
| Funded studies Enter a statement with the following details: - Initials of the authors who received each award - Grant numbers awarded to each author - The full name of each funder - URL of each funder website - Did the sponsors or funders play any role in the study design, data collection and analysis, decision to publish, or preparation of the manuscript? |  |
| Did you receive funding for this work?" |  |
| Competing Interests  Use the instructions below to enter a competing interest statement for this submission. On behalf of all authors, disclose any [competing interests](http://journals.plos.org/plosone/s/competing-interests) that could be perceived to bias this work—acknowledging all financial support and any other relevant financial or nonfinancial competing interests. | The authors have declared that no competing interests exist. |

| This statement is required for submission and will appear in the published article if the submission is accepted. Please make sure it is accurate and that any funding sources listed in your Funding Information later in the submission form are also declared in your Financial Disclosure statement.  View published research articles from [PLOS ONE](http://journals.plos.org/plosone/) for specific examples. |  |
| --- | --- |
| NO authors have competing interests Enter: The authors have declared that no competing interests exist. Authors with competing interests Enter competing interest details beginning with this statement: I have read the journal's policy and the authors of this manuscript have the following competing interests: [insert competing interests here] |  |
| * typeset |  |
| Ethics Statement  Enter an ethics statement for this submission. This statement is required if the study involved:   - Human participants - Human specimens or tissue - Vertebrate animals or cephalopods - Vertebrate embryos or tissues - Field research   Write "N/A" if the submission does not require an ethics statement.  General guidance is provided below. Consult the [submission guidelines](http://journals.plos.org/plosone/s/submission-guidelines#loc-guidelines-for-specific-study-types) for detailed instructions. Make sure that all information entered here is included in the Methods section of the manuscript. | This study was approved by the institutional ethics committee of Nepal Health  Research Council [Reg no - 2897]. As the interviews were done online, verbal consent was obtained from all the participants. |

Format for specific study types

Human Subject Research (involving human

participants and/or tissue)

Give the name of the institutional review

board or ethics committee that approved the

study

•

Include the approval number and/or a

statement indicating approval of this

research

•

Indicate the form of consent obtained

(

written/oral) or the reason that consent was

not obtained (e.g. the data were analyzed

anonymously)

•

Animal Research (involving vertebrate

animals, embryos or tissues)

Provide the name of the Institutional Animal

Care and Use Committee (IACUC) or other

relevant ethics board that reviewed the

study protocol, and indicate whether they

approved this research or granted a formal

waiver of ethical approval

•

Include an approval number if one was

obtained

•

If the study involved

non-human primates

,

add

additional details

about animal welfare

and steps taken to ameliorate suffering

•

If anesthesia, euthanasia, or any kind of

animal sacrifice is part of the study, include

briefly which substances and/or methods

were applied

•

Field Research

Include the following details if this study

involves the collection of plant, animal, or

other materials from a natural setting:

Field permit number

•

Name of the institution or relevant body that

granted permission

•

Data Availability

Authors are required to make all data

underlying the findings described fully

available, without restriction, and from the

time of publication. PLOS allows rare

exceptions to address legal and ethical

concerns. See the

[PLOS Data Polic](http://journals.plos.org/plosone/s/data-availability)

[y](http://journals.plos.org/plosone/s/data-availability)

and

[FA](http://journals.plos.org/plosone/s/data-availability#loc-faqs-for-data-policy)

[Q](http://journals.plos.org/plosone/s/data-availability#loc-faqs-for-data-policy)

for detailed information.

Yes - all data are fully available without restriction

| A Data Availability Statement describing where the data can be found is required at submission. Your answers to this question constitute the Data Availability Statement and will be published in the article, if accepted. |  |
| --- | --- |
| Important: Stating ‘data available on request from the author’ is not sufficient. If your data are only available upon request, select ‘No’ for the first question and explain your exceptional situation in the text box. |  |
| Do the authors confirm that all data underlying the findings described in their manuscript are fully available without restriction? |  |
| Describe where the data may be found in full sentences. If you are copying our sample text, replace any instances of XXX with the appropriate details.   - If the data are held or will be held in a public repository, include URLs, accession numbers or DOIs. If this information will only be available after acceptance, indicate this by ticking the box below. For example: All XXX files   are available from the XXX database (accession number(s) XXX, XXX.).   - If the data are all contained within the manuscript and/or Supporting Information files, enter the following: All relevant data are within the manuscript and its Supporting Information files. - If neither of these applies but you are able to provide details of access elsewhere, with or without limitations, please do so. For example:   Data cannot be shared publicly because of [XXX]. Data are available from the XXX Institutional Data Access / Ethics Committee (contact via XXX) for researchers who meet the criteria for access to confidential data.  The data underlying the results presented in the study are available | All relevant data are within the manuscript and its Supporting Information files. |

| from (include the name of the third party and contact information or URL).  • This text is appropriate if the data are owned by a third party and authors do not have permission to share the data.  * typeset |  |
| --- | --- |
| Additional data availability information: |  |

Manuscript	[Click here to access/download;Manuscript;Main](https://www2.cloud.editorialmanager.com/pone/download.aspx?id=38652332&guid=8e7be6db-2349-4309-bac6-e3434f161a87&scheme=1)

[Manuscript.docx](https://www2.cloud.editorialmanager.com/pone/download.aspx?id=38652332&guid=8e7be6db-2349-4309-bac6-e3434f161a87&scheme=1)

# “It becomes more difficult when people don’t empathise with us": Exploring COVID-19 stigmatisation and its ecological impact on survivors in Nepal

Buna Bhandari^1,2,3¶*^, Poshan Thapa^4¶^, Amit Timilsina^5^, Rajiv Ranjan Karn^6^, Haider Ali^7^, Ashley Hagaman,^8^ Archana Shrestha ^9^

^1^Central Department of Public Health, Tribhuvan University Institute of Medicine,

Kathmandu, Nepal

^2^Florida State University, College of Nursing, Tallassee, Florida, USA

^3^Department of Global Health and Population, Harvard T.H Chan School of Public Health,

Boston, USA

4 School of Population and Global Health, McGill University, Quebec, Canada
5 Research and Community Development Centre, IPAS, Kathmandu, Nepal
6 Department of Research, Biratnagar Eye Hospital, Biratnagar, Nepal
7 Health Division, Biratnagar Metropolitan City, Biratnagar, Nepal
8 Social and Behavioural Sciences Department, Yale School of Public Health, New Haven, USA
9 Community Health Department, Kathmandu University Dhulikhel Hospital, Dhulikhel, Nepal

^¶^ Both contributed equally

***Corresponding author** Buna Bhandari buna.bhandari@gmail.com

# Abstract

The COVID-19 pandemic has significantly impacted individuals’ psychological well-being worldwide, with stigma emerging as a critical issue for survivors. In Nepal, stigma has further complicated the experiences of survivors, influencing their health, relationships, and social interactions. This study explores the multidimensional components of COVID-19-related stigma among survivors in Eastern Nepal. This qualitative study employed in-depth interviews with 15 COVID-19 survivors, identified from a prior survey, who reported experiencing stigma. Due to pandemic-related restrictions, interviews were conducted over the phone. Data were analysed thematically, guided by the socio-ecological framework. COVID-19 stigma was multifaceted, including social rejection, self-stigmatization, discrimination by family and friends, and labelling from community members. Survivors recounted forced quarantines, financial strain, and removal from homes by police. These experiences undermined trust, selfconfidence, and mental well-being. Key drivers of stigma included fear of disease transmission, misinformation, and inadequate healthcare systems and policies that failed to address stigma effectively. Addressing COVID-19-related stigma requires a comprehensive approach, including mass education campaigns to address myths, platforms for accurate information dissemination, and strengthening health systems. Interventions must also address systemic policy and healthcare infrastructure gaps to foster an inclusive and supportive environment for survivors.

**Keywords:** COVID-19, Stigma, Discrimination, Nepal, Pandemic

# Introduction

The COVID-19 pandemic has profoundly impacted individuals’ psychological and mental well-being worldwide. Factors such as anxiety, fear, insecurity, social and emotional isolation, financial hardship, and inadequate health infrastructure have compounded the challenges faced by affected populations. Among these challenges, stigma has emerged as a critical issue for

COVID-19 survivors, exacerbating their physical, emotional, and social vulnerabilities [1,2]. Stigma experienced by survivors has been widely reported across countries, with estimates indicating prevalence rates as high as 79% in China [3], 46% in Ghana [4], and 43.3% in Japan

[5]. A pooled analysis of stigma related to infectious diseases, Severe Acute Respiratory Syndrome (SARS), Influenza A virus subtype H1N1, Middle East respiratory syndrome coronavirus (MERS), Zika virus, Ebola virus, and Coronavirus disease (COVID-19), revealed an estimated prevalence of 34%, highlighting the prevalent nature of this issue [6].

Historically, epidemics and pandemics have consistently resulted in severe social consequences, most notably stigma and discrimination [7]. This trend is evident in various infectious diseases, including Human Immunodeficiency Virus (HIV), tuberculosis (TB), SARS, Ebola, and H1N1 influenza, with COVID-19 being no exception [8]. Stigma has a detrimental impact on individuals, as demonstrated by its association with HIV. Studies have shown that HIV-related stigma contributes to depression, anxiety, and emotional distress, which in turn leads to diminished quality of life and reduced adherence to treatment protocols [9]. Consequently, understanding the stigma faced by COVID-19 survivors is crucial, given its far-reaching short and long-term consequences.

The World Health Organization (WHO) defines social stigma as the "negative association between a person or group of people who share certain characteristics and a specific disease."

Stigma manifests through labelling, stereotyping, discrimination, differential treatment, and

loss of social status. These manifestations can have severe consequences, including job termination, social exclusion, abandonment, and physical violence [10]. Furthermore, stigma drives individuals to conceal their illness, delays healthcare-seeking behaviour, and discourages the adoption of preventive measures. These repercussions extend beyond individuals, affecting families, communities, and the healthcare system. In Nepal, research on stigma related to TB, HIV, leprosy, and mental health has revealed significant impacts on patients’ health, accessibility to healthcare services, and overall quality of life [11].

Understanding the stigma faced by COVID-19 survivors is particularly crucial given increasing reports of such experiences, including in Nepal [12-13]. The nature and magnitude of stigma are highly context-specific, affecting individuals, families, communities, and societal structures at multiple levels [14]. A comprehensive understanding of stigma among COVID19 survivors can help identify its underlying drivers, develop targeted interventions, and help promote support systems. This need is especially pressing in the context of Nepal, where studies have documented stigma towards healthcare providers and migrant returnees during the pandemic [15], but few studies have explored its impact among COVID-19 survivors.

Therefore, the primary objective of this study was to explore the stigma experienced by COVID-19 survivors in Nepal. Gaining insight into their experiences can inform strategies to foster a more inclusive and supportive environment for individuals recovering from COVID-

19.

# Materials and Methods

## Study design and population

This qualitative study was conducted between May 19 and July 24, 2021, as part of a larger mixed-method research project in Biratnagar Metropolitan City, Koshi Province, Nepal. The study began with a cross-sectional survey of 385 COVID-19 survivors registered in the government reporting system. The study aimed to estimate the prevalence of mental health problems experienced by survivors. Findings revealed that 24.7% (n=101) of respondents reported experiencing some form of stigma.

For this qualitative component, survivors who reported stigma in the survey and provided informed consent for further participation were included as the sampling frame. The participants were chosen purposively to facilitate a deeper exploration of their lived experiences and the underlying factors contributing to stigma among COVID-19 survivors in Nepal.

## Data collection tool and method

To explore the stigma experiences of COVID-19 survivors, a semi-structured in-depth interview guide was developed, drawing on the socio-ecological framework. The socioecological framework which helps to understand the complex interactions between individual, interpersonal, and environmental factors contributing to COVID-19 stigma and identify ways to address multi-faceted impact of COVID-19 [16]. The interview guide included questions designed to examine stigma, its drivers, and the impact of stigma on COVID-19 survivors.

Considering the challenging circumstances of the second wave of COVID-19 in Nepal during the data collection period, all interviews were conducted over the phone. This approach was informed by previous studies that successfully utilised remote interviewing methods, balancing safety concerns, logistical feasibility, and government restrictions imposed during the pandemic. A total of 15 in-depth interviews were conducted with participants who reported experiencing some form of stigma. Research Assistants (RAs) with prior qualitative research experience and familiarity with the local context conducted the interviews. Before the data collection process, the RAs were thoroughly trained on the study objectives, interview tools, and protocols to ensure quality in data collection.

## Data analysis

The audio-recorded in-depth interviews were transcribed in the Nepali language verbatim and subsequently translated into English by the research assistant for data analysis. Translation validation was ensured by performing a back-translation of two transcripts. To ensure confidentiality, pseudonyms were assigned to participants in the transcripts.

Thematic analysis was employed to analyse the study data, following the six-step process outlined by Braun and Clarke [17]. The analysis process involved multiple stages, including familiarising with the data, generating initial codes, searching for themes, reviewing, defining, naming, and producing the final report. To enhance the rigour and reliability of the analysis, two researchers (BB/PT and AT) independently read and coded the transcripts. Inductive coding was conducted to generate data, and codes were described and grouped based on shared characteristics into sub-themes. The sub-themes were then aggregated to form overarching themes, as presented in Table 2. The researchers regularly discussed the codes, themes, and interpretations to compare and refine them. NVivo version 12 software [18] was employed to facilitate the organisation and management of the qualitative data.

## Ethical consideration

The study received ethical approval from the institutional ethics committee of the Nepal Health Research Council (Reg. No. 2897). As the interviews were conducted remotely, informed verbal consent was obtained from all participants.

# Results

## Characteristics of study participants

Table 1 summarises the demographic characteristics of the study participants who experienced stigma and discrimination. Most participants (46.6%) were aged between 31 and 45, with 53% identifying as male. Most participants (66.6%) had an education level above the higher secondary level, while 73.3% were employed. Additionally, 93% of participants reported having fewer than six family members.

**Table 1: Characteristics of study participants included in the in-depth interviews (n=15)**

| Characteristics | Categories | N (%) |
| --- | --- | --- |
| Age | 18-30 yrs. | 4 (26.6) |
|  | 31-45 yrs. | 7 (46.6) |
|  | 46 and above | 4 (26.6) |
| Sex | Female | 7 (46.6) |
|  | Male | 8 (53.3) |
| Education | Primary level | 2 (13.3) |
|  | Secondary level | 3 (20) |
|  | Higher secondary and above | 10 (66.6) |
| No of family members | 1 to 3 | 7 (46.6) |
|  | 4 to 6 | 7 (46.6) |
|  | 7 and above | 1 (6.66) |
| Occupation | Student | 3 (20) |
|  | Employed | 11 (73.3) |
|  | Unemployed | 1 (6.66) |

## Themes and sub-themes from the data

The qualitative analysis generated 53 codes, seven sub-themes, and three overarching themes. These themes highlight the diverse aspects of stigma and discrimination experienced by COVID-19 survivors. Table 2 summarises the themes, sub-themes, and corresponding codes derived from the data.

### 132 Table 2: Themes and sub-themes from the data

| **Theme** | | **Sub-themes** | **Codes** |
| --- | --- | --- | --- |
| Dimensions of multifaceted stigma |  | Stigma experience among COVID-19 survivors and their family | Internalization of stigma, perceived stigma, low family and social relationships, low family and social support, intersecting stigma, discrimination at home and work, and discrimination towards family members of COVID-19 survivors. |
|  |  | Existing stigma  practices in Nepal | Forceful quarantine, being treated as untouchable, receiving humiliating taunts, unnecessary attention, hesitance to communicate, stereotyping of COVID-19 infection and infected individuals, myths on media, and bias in healthcare seeking. |
| Impact Covid-19 stigma | of | Well-being of persons with COVID-19  infection | Limited access to medicine, lack of support for survivors, anxiety, fear of death, perceived physical weakness, mental stress, exhaustion, lack of confidence, and loneliness. |
|  |  | Fragile interpersonal trust and support | Lack of social protection, absence of caretakers at home, inability to support pregnant and postpartum women. |
| Drivers of COVID-19  stigma | | Fear of Covid-19 | Fear of contracting the virus, fear of death, inaccessible treatment, social media-perpetuated fear, high death rates, fear of recurrence, and future discrimination. |
|  |  | Limited information about  COVID-19 | Lack of knowledge regarding COVID-19, misconceptions, misinformation on social media, misunderstandings about disease transmission, lack of trust in COVID-19 tests, and ignorance regarding protocols and vaccines. |
|  |  | Fragile health system and policies | Unclear COVID-19 protocols, limited capacity of healthcare providers, lack of equipment, insufficient healthcare personnel, inadequate public health programs, lack of government preparedness, neglect of social aspects in protocols, and delayed screening and treatment. |

133

## Dimensions of multifaceted stigma

This theme highlights the various dimensions of stigma experienced by COVID-19 survivors in Nepal, focusing on their personal encounters with stigma and prevalent societal practices.

### Stigma experience among COVID-19 survivors and their family

All participants in this study reported experiencing stigma from their family and community members, manifesting in various forms and intensities during their infection. One participant linked their experience to the stigma faced by person living with HIV and Acquired Immunodeficiency syndrome (AIDS) and Tuberculosis Bacillus (TB) and shares: *There is a shopkeeper nearby, who used to tell him (neighbour who used to	take care of them) “don’t take the money”, or “don’t go near there them". This behaviour made me feel like those persons discriminated due to AIDS	or TB, which used to happen in Nepal decades ago. It did make me feel really*

*bad.* (IDI 2)

Community stigmatization was widespread once participants’ COVID-19 status was revealed. Examples included community members blocking access to homes, evicting tenants, and labelling homes and streets as *Corona ghar* or *Corona bato* ("Home infected by Coronavirus" or "Street infected by Coronavirus"). While some friends and family members were physically distant due to lockdown restrictions, a few offered support remotely. No participants reported direct discrimination from immediate family members. A female participant shared her

distress:

*I felt very discriminated... when others found out about my COVID-19 status, they stopped contacting me. When I informed them that I had tested positive, they stopped visiting my home.* (IDI 8)

Employed participants highlighted workplace stigma, including blame for disease transmission and altered interactions with colleagues, even post-recovery. Participants noted that even after testing negative, co-workers hesitated to interact with them. A female participant shared:

*Conflicts arose among the staff in our office, and they blamed each other. I had been practising self-isolation and refrained from interacting with others. Since I isolated myself early on, no one could accuse me of spreading the coronavirus to them. They would swiftly close the door when I was outside*. (IDI 3)

Participants also described stigma extending to their family members, particularly children.

Community members directed negative comments at children whose parents had contracted COVID-19, causing significant emotional distress:

*Children also faced it. When they went to buy Cetamol (medicine), people said, 'their mother is infected, why did you take that money, etc.' The child thought, 'Our mom is infected, so this money will not work,' and they became sad. They behaved discriminately against my sons.* (IDI 6*)*

Several participants expressed frustration over the lack of support from government officials and local leaders. Despite seeking assistance, some individuals were denied help and, in some cases, evicted by landlords while facing financial difficulties. One participant stated: *Some individuals who were in home isolation after testing positive for COVID-19 sought assistance from key figures in the society, including the police, but received no help. Instead, their landlords compelled them to leave their homes and had to stay at a hotel, even though they were facing financial difficulties.* (ID10)

Participants also described internalizing stigma, which affected their interactions with other COVID-19 survivors. For instance, one participant shared how fear and stigma influenced their behaviour toward their critically ill mother. One participant shared:

*Because the situation was so bad at home, everybody was scared. I was scared, too; being a son, I did not meet my mother even though she was so critical after the COVID-*

*19 infection*. (IDI 9)

## Existing Stigma Practice in Nepal

Participants described various forms of enacted stigma, including being treated as untouchables, facing humiliating remarks, and being singled out by neighbours. Stigma practices were perceived to be more pronounced in urban areas compared to rural regions. One participant shared their experience of limited support in their community:

*I didn’t get any help at the place where I reside. My friends living far away used to call, talk, and help when needed. But in the village where I live, there was no social support.* (IDI 10)

A recurring experience involved shopkeepers and cashiers refusing to handle cash directly from COVID-19 survivors, instead maintaining physical distance and discouraging others from accepting their money. One participant’s mother recounted the discrimination her children faced when attempting to purchase medicine.

*My sons went to buy medicine when I became severe. When they went to buy medicine, they said their mother was Corona positive and refused to take the money. When my sons walked in the road, they made remarks saying their mother was Corona-positive.* (IDI 7)

Another reported stigma practice involved publicly disclosing individuals’ COVID-19 status, often leading to forced quarantine at hotels, even for those facing financial hardships. Even after recovering from the disease, participants continued to face discrimination, with people hesitating to engage with them. Health workers, including doctors and nurses, faced similar experiences and could not quarantine themselves in their homes. A male participant shares: *People even refused to allow infected health workers working in hospitals as doctors or nurses, to stay in their homes. There was a widespread fear that they would transmit the virus.* (IDI 12)

Some participants with health insurance reflected on how it provided partial financial relief during their quarantine and COVID-19 treatment. However, the overall financial strain remained a significant challenge. One participant reflects:

*One thing was I had done insurance. I got the insurance money, and it did cover some expenses at that time. But it is difficult to manage things now.* (IDI 10)

## Impact of COVID-19 Stigma

### Wellbeing of the person with COVID-19 infections

Many participants reported that their physical and mental health deteriorated due to the constant discrimination and stigma they faced. Survivors experienced physical weakness from the infection, compounded by inadequate care, stress, mental exhaustion, and heightened anxiety driven by fears of reinfection and death. *[..] and at that time we had heard a lot of news saying many peoples had lost their lives in abroad because of covid and I felt like I will not be able to live now. Corona will take my life too. I had so much stress at that time which was beyond of my thinking.* (IDI 13)

Additionally, friends and community members' lack of empathetic behaviour further undermined participants’ well-being. This resulted in feelings of loneliness and a diminished sense of confidence:

*Within our family, some of us were infected while other were not infected of COVID-19. The community people suspected (falsely) that the other non- infected members were infected too. The community members used to avoid the non-infected family members while walking through our house and talk behind our back. They (the non-infected family members) felt that discrimination. No one cared to come and ask how we are dealing the situation*. (IDI 10)

### Fragile interpersonal trust and support

The lockdown imposed during the COVID-19 pandemic disrupted family connections, leaving many participants isolated from their loved ones. Migrant individuals and those away from their families expressed that the absence of family support hindered their recovery. They believed being with family could have provided the care and assistance necessary for physical and mental well-being.

*Maybe it's because I didn’t get any support then. I was far from my family during that COVID-19 situation; I think that if my family was there, they could have helped me get medicine whenever I needed it*. (IDI 14)

Discrimination and stigma also led to severe consequences, including the death of individuals due to a lack of community support and resources. One participant recalled a neighbour who passed away because he could not receive even the most basic care:

*There was a lot of discrimination. Near to my home, due to lack of care, even he was unable to boil water by himself. At the time of taking him to a hospital, he died.* (IDI-

12)

## Drivers of COVID-19 stigma

### Fear of COVID-19 transmission and death

The fear of contracting the virus and dying from it was one of the most commonly cited reasons for stigma. Participants highlighted how the lack of guaranteed treatment or vaccines during the early phase of the pandemic contributed to fear and avoidance of COVID-19 survivors:

*Fear!! People had limited knowledge about the disease, its transmission, and its causes. They were afraid of the COVID-19 outbreak, and there was a lack of understanding about disease prevention.* (IDI 10)

Participants also described the fear of being forcibly quarantined by authorities, which led many to hide their COVID-positive status, disclosing it only to close family members: *During that time, the main problem was that the people were scared of being taken by the police and used to hide the fact that they had the disease. If any case were found,*

*the area would have been sealed, people would have hesitated to go near the patients, and even the whole family would have faced the same.* (IDI 10)

Social media played a significant role in amplifying stigma, often sensationalizing and misrepresenting isolation practices, which negatively impacted survivors:

*At the time of being diseased, people are mentally weak and left alone. Social media posts about people in isolation affected patients mentally. It was unpractical and unscientific.* (IDI 14)

### Limited Information regarding COVID-19

Participants attributed stigma to widespread misinformation and a lack of awareness about COVID-19 transmission, testing, and preventive measures. Many individuals held misconceptions, such as believing that COVID-19 survivors would inevitably die or that the virus remained in the body despite a negative test result:

*Corona gets transmitted by not wearing a mask properly, staying close, or handshaking. People should understand these things to stop discriminating against COVID-19 patients.* (IDI 7)

The lack of awareness contributed to discriminatory behaviours, with some participants expressing shame and fear of social interactions due to misinformation:

*Discrimination occurs due to a lack of awareness, nothing more than that. Instead of discrimination, safety measures should be in place, such as maintaining distance. It's okay not to hug or be too close. Initially, I felt shy and fearful when facing others, fearing negative comments. Even after receiving a negative test report, I still had a lingering fear. There was a lack of awareness in the community, and people remain uninformed.* (IDI 12).

Participants stressed the importance of adhering to protocol and guidelines and highlighted the need of social and behavioural change communication programs, community advocacy, and mobilisation of health volunteers to address these gaps.

*Had society been well-informed and educated, such discrimination wouldn’t have occurred. I know ummm, for a disease like corona (COVID-19), it is necessary to keep some distance, but there is a standard protocol of 3 metres or 6 metres. All of that happened because of a lack of proper information.* (IDI 2)

*Awareness campaigns should run so swiftly that all should understand this disease. It could also be about vaccination. I have gotten my vaccination, but many haven’t; even*

*for that, awareness is a necessity.* (IDI 1)

### Fragile health system and policy

Participants frequently cited inadequate health infrastructure as a significant driver of stigma. Limited hospital beds, a shortage of equipment, and untrained healthcare workers compromised COVID-19 care and fuelled stigma:

*Even the patients are not getting beds in the hospital after being in the emergency for a long time. I don’t think there was any support from the community health system or government.* (IDI 14)

*The fear of infection and the lack of guaranteed treatment at a health facility made the discrimination worse.* (IDI 2)

Participants highlighted the government's lack of preparedness in responding to the pandemic. Issues included inadequate enforcement of COVID-19 protocols, ineffective communication of guidelines, and a lack of concrete plans to address myths and misconceptions. One participant recounted:

*We know everything and have a lockdown for prevention, but it still lacks safety measures. Our government is lacking in maintaining safety. Talking about my experience, I returned home from Kathmandu the last time before this second lockdown (second wave). At that time, it was said that vehicles were running under safety measures, maintaining distance, and sanitising, but it was not like that. There were as many passengers as before when COVID was not introduced without maintaining social distancing. It was so packed, and bus fare was also double or triple the regular amount. And that’s not how to do it; it was not satisfying.* (IDI 4)

A widespread lack of trust in the government was evident among participants, who expressed dissatisfaction with its response and strategies. Participants also pointed out the gap between the development and implementation of protocols, which led to increased myths and misconceptions. One participant shares:

*Government did nothing; everything is just empty promises and lectures. It’s just written on paper. Everything is just based on books and paper, so we should not expect from the government and say that the government didn’t do this or that. We ourselves should be aware.* (IDI 3)

## Recommendations for future preparedness

Participants suggested that the government prioritise adequate preparedness and planning for future pandemics. This includes developing policies, strategies, tools, and procedures to address multi-dimensional stigma. A participant emphasised the importance of social discrimination policies:

*Policy about social discrimination should be prepared. It should provide justice for the affected people in a time of need. I feel like COVID-19 will remain for a long time, like tuberculosis in Nepal, but the cases can be decreased, and some COVID hospitals will continue to function.* (IDI 15)

Apart from this, the participants also suggested that the government improve its preparedness by learning from COVID-19 and ensure social security provision, improved financial aid, supplement packages, nutrition food, and provision of essential medicines during the pandemic to normalise the fear of pandemic and strengthen the health system.

*The government should look after the coronavirus victims from a low financial background and help them with financial support and supplementation. Some financially unstable coronavirus victims have to fight against such disease with just a meal of dal bhat (rice and lentils), so a little help there in a proper supplement package and nutrition or through direct financial support would be better*. (IDI 5)

# Discussion

This qualitative study provides valuable insights into the multifaceted experiences of stigmatisation during the COVID-19 pandemic. It explores various forms of stigma, including personal experiences, societal practices, impacts, drivers, and potential solutions to address COVID-19-related stigma. The findings particularly emphasise the enacted and associated stigma faced by COVID-19 survivors, leading to interpersonal discrimination from neighbours and community members. Additionally, structural and systemic discrimination was perpetuated by the lack of comprehensive policies, clear guidelines, effective implementation of COVID-19 protocols, and a fragile health system.

Similar patterns of stigma and discrimination toward COVID-19 patients have been documented in other countries [19-21]. For instance, studies from India have reported high levels of enacted and internalised stigma among COVID-19 survivors [20]. Similarly, research conducted in China has highlighted significant stigma experienced by COVID-19 patients, mainly manifesting as social rejection, financial insecurity, internalised shame, and social isolation, factors that align closely with the findings of this study [21].

Our study participants reported various forms of enacted stigma, including being treated as untouchables, enduring humiliating remarks, and having their residential areas labelled as

"Corona home" or "Corona Street." Families of COVID-19 survivors also faced social rejection and discriminatory behaviours. Similar patterns of labelling, discrimination against family members, and social rejection have been documented in studies conducted in Uganda and Jordan [22, 23]. Additionally, some studies have reported more severe forms of stigma, such as physical violence and verbal insults [20, 24-25]. However, it is reassuring that participants in our study did not encounter such extreme manifestations of stigma.

Consistent with the findings of Regmi et al., this study reports lower discriminatory behaviours from family members and healthcare workers. This is an encouraging observation, as these groups can provide essential emotional and practical support to COVID-19 survivors [26]. However, stigmatisation and discrimination significantly impacted the physical health, mental health, and overall well-being of participants in this study, aligning with findings from other global studies [27]. The reasons behind stigma and discrimination identified in this study are multifaceted and context specific. Fear of disease transmission, recurrence, and death emerged as primary drivers of stigma. Additionally, misinformation, inadequate information, and a lack of awareness about COVID-19 transmission, management, and prevention were key contributing factors. The prevalence of misinformation, mainly through digital platforms, has been well-documented during the COVID-19 pandemic [28-30]. Addressing these information gaps should be a priority for authorities, particularly in low- and middle-income countries (LMICs), where the impact of misinformation can be more pronounced.

One of the unique findings of this study is the connection between a fragile health system and policy gaps with the stigmatisation of COVID-19 survivors. Participants highlighted several systemic shortcomings that contributed to stigma, including the lack of comprehensive plans and policies to address the social aspects of COVID-19, insufficient capacity among healthcare workers, inadequate social and behaviour change communication for effective implementation of COVID-19 guidelines, and a general lack of preparedness across all tiers of government.

Additional challenges included fragile health infrastructure, delayed vaccination efforts, and the absence of platforms to counter myths and misconceptions. These factors collectively contributed to the stigmatisation of COVID-19 survivors and multifaceted discrimination. Participants also perceived government protocols as a source of discrimination, highlighting policy design and implementation gaps. This aligns with findings from multiple studies, which have reported that fragile health systems, inadequate governmental preparedness, and a lack of accurate information were major contributors to stigma during the pandemic [22, 25,31-32].

Urgent actions are required to prevent and mitigate infectious disease-related stigma, particularly in LMICs [33]. Mass education and awareness campaigns are essential to improve public knowledge about disease transmission, prevention, and control, not only for COVID-19 but also for future emerging infectious diseases. There is also a critical need to establish clear policies, regulations, and reporting systems to strengthen fragile health system.

## Strengths and limitations

A key strength of this study is its exploration of the multi-dimensional nature of COVID-19

stigmatisation using a socio-ecological framework. This approach allowed for a comprehensive examination of the stigma, its drivers and impacts from multiple lenses, addressing gaps left by previous research. The study findings provide valuable guidance for policymakers, government officials, and community stakeholders to integrate socio-ecological approaches into strategies for addressing stigma during humanitarian crises such as COVID-19. Additionally, the study included participants from diverse backgrounds which facilitated a thorough understanding of the stigma experienced by survivors across various demographic groups.

The study also has a few limitations. First, data collection was conducted through virtual interviews, which may have restricted the ability to observe and capture the non-verbal cues in participants' responses. Second, the self-reported nature of the data introduces potential recall and social desirability biases, as participants may not fully or accurately recount their experiences of stigma. To address these limitations, the study employed a rigorous methodological approach, including developing and pretesting the interview tool, comprehensive training of research assistants, and using probing techniques during interviews to capture detailed responses. Additionally, to minimise researcher bias, two researchers conducted qualitative data coding independently, and discrepancies were resolved through discussion and consensus. Finally, the findings are specific to the context of Biratnagar Metropolitan City in Nepal and may not be transferable to other regions or populations.

# Conclusion

This study sheds light on the significant issue of stigma experienced by COVID-19 survivors in Nepal. The findings reveal the multi-level and multifaceted nature of stigma, encompassing social rejection, self-perceived stigma, discrimination by family and friends, and negative labelling from community members. Participants reported being forced into hotel quarantine despite financial hardships and being removed by the police, further eroding trust, diminishing confidence, and adversely affecting their physical and mental health and overall well-being. The drivers of stigma and discrimination identified in this study include a lack of knowledge and awareness about COVID-19 infection, screening, and treatment; fear of infection and its consequences; a fragile healthcare delivery system; incomprehensive policies; and inadequate strategies to address myths and misconceptions. These findings emphasize the urgent need for comprehensive interventions to combat stigma and discrimination. The key recommendations include implementing mass education and awareness campaigns to improve knowledge about disease transmission, prevention, and control, establishing platforms for accessing accurate information, strengthening reporting systems, enhancing the capacity of health institutions, and developing robust social protection mechanisms to support individuals facing stigma.

# Acknowledgment

We would like to acknowledge the health section of the Biratnagar Metropolitan City Office team for providing data, all the Research Assistants who helped in data collection, transcription, and translation, and the Nepal Health Research Council for the financial support

to conduct this study.

# References

1. Chaudhary U. COVID-19 Sharpens Caste Discrimination in Nepal: Inter Press Service News Agency 2020 [cited 2020 28 September ]. Available from:

<https://nepalitimes.com/here-now/covid-19-sharpens-caste-discrimination-in-nepal>

2. Parajuli S. Say no to coronavirus stigmatisation: The Kathmandu Post 2020 [cited 2020 September 28]. Available from: [https://kathmandupost.com/columns/2020/08/02/sayno-to-coronavirus-stigmatisation.](https://kathmandupost.com/columns/2020/08/02/say-no-to-coronavirus-stigmatisation)
3. Liu R, Nicholas S, Leng A, Qian D, Maitland E, Wang J. The influencing factors of

452 discrimination against recovered Coronavirus disease 2019 (COVID-19) patients in 453

China: a national study. Human Vaccines & Immunotherapeutics. 2022 Jan

454

31;18(1):1913966.

455

4.	Osei E, Amu H, Appiah PK, Amponsah SB, Danso E, Oppong S, et.al. Stigma and

456 discrimination tendencies towards COVID-19 survivors: evidence from a nationwide
457 population-based survey in Ghana. PLOS global public health. 2022 Jun

458

22;2(6):e0000307.

459

5. Sugiyama A, Miwata K, Kitahara Y, Okimoto M, Abe K, E B, Ouoba S, et.al. Long 460

COVID occurrence in COVID-19 survivors. Scientific reports. 2022 Apr 461

11;12(1):6039. 462

6. Yuan K, Huang XL, Yan W, Zhang YX, Gong YM, Su SZ, et.al. A systematic review

463 and meta-analysis on the prevalence of stigma in infectious diseases, including COVID-

464

19: a call to action. Molecular psychiatry. 2022 Jan;27(1):19-33.

465

7.	Craddock S. Sewers and scapegoats: spatial metaphors of smallpox in nineteenth

466 century San Francisco. Social science & medicine. 1995 Oct 1;41(7):957-68.

467

8.	Chang SH, Cataldo J. A systematic review of global cultural variations in knowledge,

468 attitudes and health responses to tuberculosis stigma. The International Journal of

469

Tuberculosis and Lung Disease. 2014 Feb 1;18(2):168-73.

470

9.	Rueda S, Mitra S, Chen S, Gogolishvili D, Globerman J, Chambers L, et.al. Examining

471 the associations between HIV-related stigma and health outcomes in people living with

472

HIV/AIDS: a series of meta-analyses. BMJ open. 2016 Jul 1;6(7):e011453. 473

474 10.	A guide to preventing and addressing social stigma associated with COVID-19.
475 COVID-19: Risk communication and community engagement. February 24, 2022.
476 [https://www.who.int/publications/m/item/a-guide-to-preventing-and-addressing-](https://www.who.int/publications/m/item/a-guide-to-preventing-and-addressing-social-stigma-associated-with-covid-19)
477 [social-stigma-associated-with-covid-19](https://www.who.int/publications/m/item/a-guide-to-preventing-and-addressing-social-stigma-associated-with-covid-19)
    11. Marahatta SB, Amatya R, Adhikari S, Giri D, Lama S, Kaehler N, et.al. Perceived
478 stigma of leprosy among community members and health care providers in Lalitpur
479 district of Nepal: A qualitative study. PLoS One. 2018 Dec 27;13(12):e0209676.

480

12. Singh R, Subedi M. COVID-19 and Stigma: Social discrimination towards frontline

481 healthcare providers and COVID-19 recovered patients in Nepal. Asian J Psychiatr.

482

2020 53:102222.

483

13.	Bhattacharya P, Banerjee D, Rao TS. The “Untold” Side of COVID-19: Social Stigma

484 and Its Consequences in India. Indian Journal of Psychological Medicine.

485

2020;42(4):382-6.

486

14.	Stangl AL, Earnshaw VA, Logie CH, van Brakel W, C. Simbayi L, Barré I, et al. The 487

Health Stigma and Discrimination Framework: a global, crosscutting framework to

488 inform research, intervention development, and policy on health-related stigmas. BMC
489 medicine. 2019;17(1):31.

490

15.	Singh R, Subedi M. COVID-19 and stigma: Social discrimination towards frontline

491 healthcare providers and COVID-19 recovered patients in Nepal. Asian journal of
492 psychiatry. 2020 Oct;53:102222.

493

16. Kilanowski JF. Breadth of the socio-ecological model. Journal of agromedicine. 2017

494

Oct 2;22(4):295-7.

495

17. Braun V, Clarke V. Using thematic analysis in psychology. Qualitative research in

496 psychology. 2006;3(2):77-101.

497

18.	QSR International (1999) NVivo Qualitative Data Analysis Software [Software].

498

[Internet]. Available from: Available from [https://qsrinternational.com/nvivo/nvivo-](https://qsrinternational.com/nvivo/nvivo-products/)

499 [products/.](https://qsrinternational.com/nvivo/nvivo-products/) 500

19.	Muhidin S, Vizheh M, Moghadam ZB. Anticipating COVID-19-related stigma in

501 survivors and health-care workers: Lessons from previous infectious diseases outbreaks

502

- An integrative literature review. Psychiatry Clin Neurosci. 2020;74(11):617-8.

503

20. Bhanot D, Singh T, Verma SK, Sharad S. Stigma and Discrimination During COVID-

504

19 Pandemic. Frontiers in public health. 2021;8:829.

505

21. Yuan Y, Zhao Y-J, Zhang Q-E, Zhang L, Cheung T, Jackson T, et al. COVID-19-related

506 stigma and its sociodemographic correlates: a comparative study. Globalization and

507

Health. 2021;17(1):54.

508

22. Amir K. COVID-19 and its related stigma: A qualitative study among survivors in

509

Kampala, Uganda. Stigma and Health. 2021.

510

23. Abuhammad S, Alzoubi KH, Khabour O. Fear of COVID-19 and stigmatization
511 towards infected people among Jordanian people. International journal of clinical
512 practice. 2021;75(4):e13899.

513

24.	Dar SA, Khurshid SQ, Wani ZA, Khanam A, Haq I, Shah NN, et al. Stigma in

514 coronavirus disease-19 survivors in Kashmir, India: A cross-sectional exploratory
515 study. PloS one. 2020;15(11):e0240152.

516

25. Bagcchi S. Stigma during the COVID-19 pandemic. The Lancet Infectious Diseases. 517

2020;20(7):782. 518

26. Regmi PR, Dhakal Adhikari S, Aryal N, Wasti SP, van Teijlingen E. Fear, stigma and

519 othering: The impact of COVID-19 rumours on returnee migrants and muslim
520 populations of Nepal. International Journal of Environmental Research and Public

521

Health. 2022 Jul 23;19(15):8986.

522

27.	Cervantes-Guevara G, Maciel-Saldierna M, Elizondo-Hernandez E, Cervantes-Perez 523

LA, Cervantes-Cardona GA, Garcia-Reyna B, et al. Fear of COVID-19 in high school

524 personnel: A survey study in Western Mexico. International Journal of Mental Health
525 and Addiction. 2022;20(3):1687-94.

526

28.	Love JS, Blumenberg A, Horowitz Z. The Parallel Pandemic: Medical Misinformation

527 and COVID-19. Journal of General Internal Medicine. 2020;35(8):2435-6.

528

29.	Ecker UKH, Lewandowsky S, Cook J, Schmid P, Fazio LK, Brashier N, et al. The

529 psychological drivers of misinformation belief and its resistance to correction. Nature

530

Reviews Psychology. 2022;1(1):13-29.

531

30. Khullar D. Social Media and Medical Misinformation: Confronting New Variants of an

532

Old Problem. JAMA. 2022;328(14):1393-4.

533

31. Sadati AK, Parvizi MM, Forouhari S, Hosseini SA, Jahromi MH, Jafferany M. A

534 qualitative study on stigmatization associated with COVID-19. The Primary Care

535

Companion for CNS Disorders. 2022 Mar 24;24(2):40320.

536

32.	Bhatt N, Bhatt B, Gurung S,Dahal S, Jaishi AR, Neupane B et al*.* Perceptions and

537 experiences of the public regarding the COVID-19 pandemic in Nepal: a qualitative

538

study	using	phenomenological	analysis.	*BMJ*

539

*Open* 2020;**10:**e043312. doi: 10.1136/bmjopen-2020-043312

540

33.	Yuan K, Huang X-L, Yan W, Zhang Y-X, Gong Y-M, Su S-Z, et al. A systematic

541 review and meta-analysis on the prevalence of stigma in infectious diseases, including

542

COVID-19: a call to action. Molecular Psychiatry. 2021.

543

544

545	546

547
